# Internal Derangements of the Knee-Joint

**Published:** 1909-05-15

**Authors:** 


### SURGERY.

### INTERNAL DERANGEMENTS OF THE KNEE-JOINT.

The knee-joint is more exposed to injury than any other joint in the body. The assumption of the erect attitude in man has caused modifications in its anatomical structure. In order to support the body, the articular ends of the bones which enter into its formation are large and broad; and in order to allow free flexion and extension, as in walking, or more particularly in running, the bulk of the periarticular structures has been reduced to a minimum; and the joint is surrounded not by muscles, but by tendons. It is, therefore, peculiarly liable to be injured by blows from without, and has also little power of resisting any strain thrown upon it by a lateral twist.

As a result of such strains it is common to find that some portion of the delicate internal mechanism of the joint is displaced or deranged. At one time all such injuries were classed under the heading of "loose cartilage"; but it is now recognised that there is a large variety of injuries occurring in the knee-joint which give rise to a similar series of symptoms, in some of which the cartilage itself is neither damaged nor loose, and it is therefore convenient to employ the more comprehensive title "internal derangements of the knee-joint," which covers all.

The semilunar cartilage may be damaged and become swollen, as the result of a strain, without its necessarily being detached, and this may lead to some chronic thickening of its anterior cornu, which may be pinched when the joint is extended, and so impair the freedom of movement. Or, again, the tip of the cartilage only may be detached and twisted on itself with a similar result. Much more rarely the whole cartilage is torn from its attachments and forms a genuine loose body within the joint. And the cartilage may be torn across in the longitudinal or transverse axis, the two halves remaining attached to the bone.

A genuine loose body in the joint may cause symptoms almost identical with those which are associated with a torn semi-lunar cartilage. The causes of a loose body are legion. They may result from the detachment of one of the normal structures—*e.g.* a semi-lunar cartilage or some part of it; but quite as often one of the accessory structures, such as the ligamenta alaria, is the starting point of the trouble. But the most frequent cause of a genuine loose body is the detachment of outgrowths which are not normally present, but owe their existence to a pathological condition of the joint. Such are the cartilaginous processes which are formed upon the synovium or upon the semilunar cartilages themselves in osteo-arthritis or the so-called "melon-seed" bodies which are occasionally the outcome of altered blood clot, but are more commonly tuberculous in origin.

But whether the condition results from normal or abnormal constituents of the joint, it is always, or nearly always, directly caused by a lateral twist or strain; and football and tennis are the most fertile cause of such accidents. After the accident the patient is conscious of great pain in the knee-joint, and has to keep to his bed. In a short time, generally a few hours, but at most a day or two, the knee begins to swell up on account of the accompanying synovitis with effusion. This subsides in a varying time, and when he first begins to get about again the patient is conscious only of a sense of weakness in the affected joint. The weakness is due to a residual chronic synovitis, the inflammatory exudate being rarely, if ever, completely absorbed so as to leave the joint in as good a condition as it was before. In addition to this, if there has been much fluid in the joint, the capsule and the periarticular structures will have been definitely weakened by the increased tension to which they have been subjected, even if only for a comparatively short time. A knee-joint which has once suffered acute trauma is rarely quite sound again.

The patient soon becomes conscious of this weakness, because the knee exhibits a tendency to swell after any strain has been thrown upon it, as in walking or more particularly in standing still for any length of time.

If there is a loose body in the joint other and more serious results ensue. The loose structure occasionally gets nipped between the ends of the bones, causing an excruciating pain, so that the patient often falls to the ground with such suddenness that he is unable to protect himself by putting out his hand: moreover the knee is often found locked after such occurrences, that is to say the leg is found to be flexed at the knee-joint and cannot be actively extended, so that traction has to be applied. After each such occasion there is a recurrence of the synovitis, which lasts for a few days.

When the knee is examined in the quiet stage it is quite usual to find no gross evidence of any damage. Its outline is normal and movements may be easily and naturally performed; but occasionally there is a distinct click when the leg is extended, and more rarely a loose body can be felt to be extruded laterally from the joint. The patient can sometimes make it appear voluntarily.

The treatment of this condition is not very satisfactory. It may be laid down as a rule that all palliative measures should be tried before operation is resorted to. The best results are obtained by massaging the leg and making the patient do exercises. An efficient exerciser for home use may be made with a stirrup attached to a tin of shot by a rope, which is passed over a pulley at a convenient distance from the ground. The foot is placed in the stirrup and works up and down against the resistance of the weight. The rationale of this treatment is to increase the strength of the capsule and periarticular structures, so that the loose body is kept within due bounds. The removal of the loose body by operation gives brilliant results in selected cases, and is specially indicated in those in which a loose body, whether it be a cartilage or synovial fringe can be felt. It is also demanded of the recurrent attacks of nipping of the loose body with synovitis and locking of the joint are so frequent as to incapacitate the patient from pursuing his occupations. But if these operations are performed indiscriminately many disappointing results will be encountered.

